# Photo-oxidative degradation of polyacids derived ceria nanoparticle modulation for chemical mechanical polishing

**DOI:** 10.1038/s41598-021-03866-9

**Published:** 2022-01-31

**Authors:** Eungchul Kim, Jiah Hong, Hyunho Seok, Taesung Kim

**Affiliations:** 1 School of Mechanical Engineering, Sungkyunkwan University, Suwon, 16419 South Korea; 2 SKKU Advanced Institute of Nanotechnology (SAINT), Sungkyunkwan University, Suwon, 16419 South Korea

**Keywords:** Polymer chemistry, Mechanical engineering, Nanoparticles

## Abstract

The effects of photo-oxidative degradation of polyacids at various concentrations and with different durations of ultraviolet (UV) irradiation on the photo-reduction of ceria nanoparticles were investigated. The effect of UV-treated ceria on the performance of chemical mechanical polishing (CMP) for the dielectric layer was also evaluated. When the polyacids were exposed to UV light, they underwent photo-oxidation with consumption of the dissolved oxygen in slurry. UV-treated ceria particles formed oxygen vacancies by absorbing photon energy, resulting in increased Ce^3+^ ions concentration on the surface, and when the oxygen level of the solution was lowered by the photo-oxidation of polymers, the formation of Ce^3+^ ions was promoted from 14.2 to 36.5%. Furthermore, chain scissions of polymers occurred during the oxidation process, and polyacids with lower molecular weights were found to be effective in ceria particle dispersion in terms of the decrease in the mean diameter and size distribution maintaining under 0.1 of polydispersity index. With increasing polyacid concentration and UV irradiation time, the Ce^3+^ concentration and the dispersity of ceria both increased due to the photo-oxidative degradation of the polymer; this enhanced the CMP performance in terms of 87% improved material removal rate and 48% lowered wafer surface roughness.

## Introduction

Chemical mechanical polishing (CMP) is an effective technique to achieve local and global planarization of semiconductor wafers for the development of next-generation devices. With recent developments in three-dimensional integrated circuits of semiconductor devices, such as NAND flash memory, the number of vertically stacked gates has significantly increased and larger step heights are encountered^1–3^. Therefore, it is crucial to meet the requirements of a high oxide removal rate to overcome these new challenges for the CMP process. Ceria is known to exhibit a high oxide removal rate due to its strong chemical interaction with an oxide surface^4,5^. Cook first proposed the “chemical tooth” model to explain the removal mechanism of ceria abrasives used to obtain a high polishing efficiency for glass substrates^6^. Several mechanisms for the same have been suggested by many different researchers^7–9^ and it is widely known that active sites for reactions consist of Ce^3+^ ions on the ceria surfaces^10–12^. The formation of an oxygen vacancy results in the reduction of cerium ions in the lattice from Ce^4+^ to Ce^3+^^13–15^. Ce^3+^ plays a vital role in determining the reaction with the oxide surface, forming strong Ce-O-Si bonds^16–18^. It has been reported that a higher surface concentration of Ce^3+^ ions leads to a higher removal rate of the SiO_2_ layer due to the strong interaction between ceria and SiO_2_^19,20^. Several studies have been conducted to improve the oxide removal efficiency by increasing the Ce^3+^ concentration in ceria abrasives. Kim et al. investigated the effect of the concentration of Ce^3+^ ions on the removal rate of SiO_2_ layers and reported a method to synthesize ceria particles with a high concentration of Ce^3+^ ions by reducing the primary particle size^21^. Wang et al. found that the ratio of Ce^3+^ to Ce^4+^ ions on the abrasive surface increases with decreasing concentration of ceria slurry, which results in improved removal rates in optical glass CMP^22^. Various approaches have been proposed to enhance the catalytic activity of ceria by promoting surface oxygen vacancy formation. Several studies have shown that doping metal ions into CeO_2_ lattice results in superior catalytic performance by lowering the oxygen vacancy formation energy required to detach an oxygen atom from the surface^23,24^. Previously, we demonstrated that UV light irradiation can generate a large number of oxygen vacancy defects and increase the Ce^3+^ concentration, leading to an effective reduction of ceria^25^. It is known that ambient oxygen level promotes a reoxidation process of ceria, in other words, inhibits the reduction reaction of ceria^26^. Therefore, a low oxygen level is highly desirable for enhanced oxygen vacancy formation because this condition prevents the reoxidation of ceria particles by oxygen in the ambient environment.

In this study, photo-oxidative degradation of polymers was adopted to lower the oxygen level in ceria slurry. A few water-soluble polymer chains undergo photo-oxidative reactions under the exposure of UV light, which leads to chain scission^27^. Polyacids used as dispersing agents in ceria slurries, such as poly(acrylic acid) (PAA) and poly(methacrylic acid) (PMA), are degraded under the exposure of UV light and a low-oxygen environment is achieved by the photo-oxidation process. In addition, the degraded polymers with low molecular weights increase the interaction between particles. Therefore, this improves the dispersibility of ceria particles.

Herein we propose a new strategy to improve the oxide CMP performance by photo-degradation of polyacids, which accelerates oxygen vacancy formation of ceria abrasives under UV-light irradiation. The photocatalytic degradation behavior of polymers in ceria slurry was studied, and its effect on the concentration of Ce^3+^ ions and CMP performance was evaluated.

## Results and discussion

### Photo-oxidation of polyacids

UV light enhances the physical reactivity of ceria. UV light reduces ceria by forming oxygen vacancies on its surface. The oxygen vacancy formation energy on the ceria surface is 1.16 eV and the separation of the electron-hole pair upon UV light irradiation decreases the oxygen vacancy formation energy to a negative value (− 0.64 eV)^28,29^. Therefore, UV light irradiation on the ceria is directly involved in the creation of oxygen vacancy. The electrons and holes generated from the UV irradiation reduce Ce^4+^ to Ce^3+^ and the holes oxidize oxygen anions. Since ceria releases oxygen in the process of reduction, it should be reacted at a low oxygen level to efficiently reduce ceria^26^. Since dissolved oxygen can oxidize ceria, the process of removing dissolved oxygen is necessary to prevent reoxidation of ceria by extracted oxygen from it^30^. In this experiment, the photo-oxidation reaction of polyacids including PAA and PMA was induced to remove dissolved oxygen during the reduction of ceria by UV irradiation. PAA and PMA are anionic surfactants with negatively charged carboxylic groups in the main chain and are commonly used as dispersants to enhance the dispersibility of particles upon adsorption to ceria surfaces with positive potential in acidic media^31–35^. Polyacids undergo photo-oxidation and photo-degradation when exposed to oxygen and UV light. UV irradiation on polyacids causes cleavage of chemical bonds in main chains and side-groups, as shown in the following Reactions (1–3)^36–38^.

4 $$  {\text{P}}^{*}  + {\text{O}}_{2} \xrightarrow{{{\text{hv}}}}{\text{POO}}^{*}   $$

The same macroradicals are created in PMA and PAA. Following photo-oxidation causes the polymer chains to break, and this is known as chain scission^39–41^. In the presence of oxygen, polyacids exposed to UV light react with oxygen to form macroperoxyradicals as shown in Reaction (4)^42^.

Macroperoxyradicals can be propagated in a series of reactions that follow free radical mechanisms^38^. In general, the photooxidative degradation of polymers follows the Reactions (5–9) with chain scission^43^.

5 $$  {\text{POO}}^{*}  + {\text{PH}}\xrightarrow{{{\text{hv}}}}{\text{POOH}} + {\text{P}}^{*}   $$

6 $$ {\text{POOH}}\to ^{{ }} {\text{ PO}}^{*} + {\text{HO}}^{*} $$

7 $$ 2{\text{POOH}}\to ^{{ }} {\text{ POO}}^{*} + {\text{PO}}^{*} + {\text{H}}_{2} {\text{O}} $$

8 $$ {\text{PO}}^{*} + {\text{PH}}\to ^{{ }} {\text{ POH}} + {\text{P}}^{*} $$

9 $$ {\text{HO}}^{*} + {\text{PH}}\to ^{{ }} {\text{ H}}_{2} {\text{O}} + {\text{P}}^{*} $$

**Figure 1:**
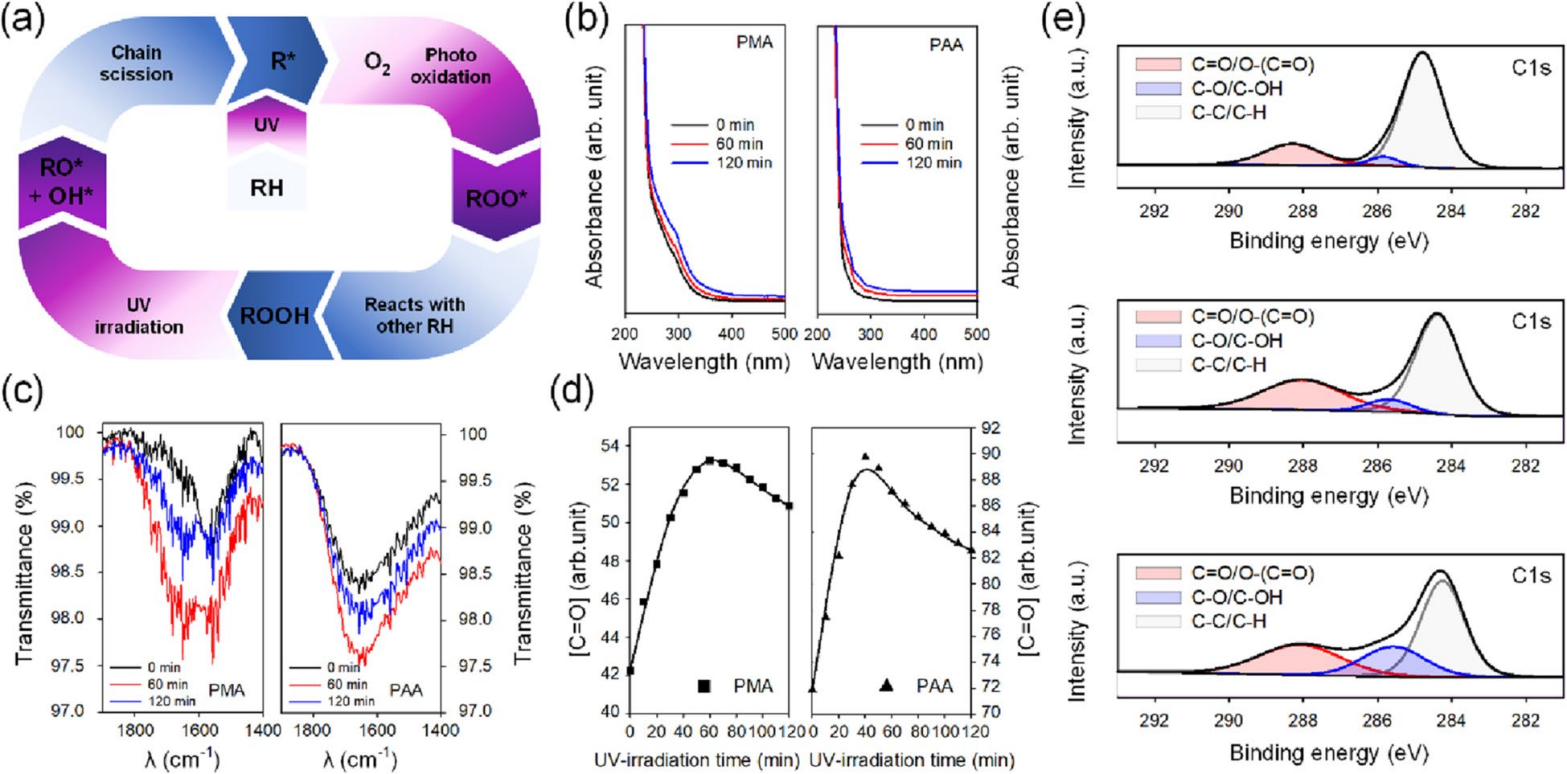
(**a**) General oxidation and photo-oxidation cycle of polymers. (**b**) Absorbance spectra and (**c**) FTIR spectrum of UV irradiated PMA (left) and PAA (right) by the UV irradiation time; 0, 60, and 120 min. (**d**) Changes in integral intensity of carbonyl band (1707 cm^−1^) during UV irradiation for 120 min. (**e**) XPS spectra of PAA according to UV irradiation time; (top) 0 min, (middle) 60 min, and (bottom) 120 min. The peak at 288.2 eV is a C = O bonding and the 285.8 eV peak indicates C-O bonding. The peak at 284.4 eV is corresponding to C–C and C-H of the polymer chain^47–49^.

This process can be repeated in a loop, as shown in Figure 1a, until the energy is continuously applied, and the amount of material is sufficient. The formation of HO* radical during UV irradiation is very efficient, and it is the main reason for the accelerated decomposition of both polymers^38,42,43^. The radicals formed during polymer degradation can be terminated by various mixtures containing different functional groups, such as aldehyde, ketone, ester, peracid, alcohol, peroxide, and double bonds.

The absorption spectra of chromophores containing ethylene bonds and carbonyl groups, which were produced by the photo-oxidative degradation of polyacids, depend on their concentrations. Figure 1b shows an increase in the absorbance at 280 and 300 nm due to chromophore formation in UV-irradiated PMA and PAA with increasing duration of irradiation, implying that the photoreaction of polyacids was accelerated with increasing duration of UV irradiation^44–46^.

The photo-oxidation processes were monitored by FTIR spectroscopy. Owing to the high absorption capacity of water by polyacids, the band in the hydroxy group (3000–3600 cm^−1^) was not considered. A significant peak at 1707 cm^−1^ was observed, which attributed to the C=O stretching vibration of the carbonyl group, as shown in Fig. 1c. The carbonyl band of polyacids verifies photo-oxidative degradation. The integral intensity of carbonyl groups increases rapidly and decreased gradually when the number of carbonyl groups reached a maximum value, as shown in Fig. 1d. The change in maximum absorbance of a carbonyl group with increasing duration of UV irradiation shows that an opposite reaction occurred, which involved the destruction of carboxylic groups and oxidation of macro-chain. At the beginning of UV irradiation, several new carbonyl groups were formed by the photo-oxidation process, leading to a rapid increase in intensity. However, it decreased slightly due to the decomposition of carboxyl groups as the dissolved oxygen levels were lowered and chain scission was promoted by the macroperoxyradical. According to the XPS spectra shown in Fig. 1e, most of the chromophores formed from photo-oxidation of polyacids are defined as C=O bonds of a carbonyl group along with FTIR results. In the case of the pristine PAA, the majority of peaks exist for C–C and C–H of the chain backbone and C=O bonding contained in some carboxyl groups^47^. In the PAA subjected to UV treatment for 60 min, the ratio of C=O bonding from the carbonyl group increased due to oxidation, and at the same time, the C–O bands slightly increased due to hydroxyl group and alkoxy radicals generated during the degradation process^48,49^. After 120 min, because the degradation process is promoted compared to the oxidation process, the intensity of C–O bands increases significantly with a slight decrease in the portion of the C=O peak. Detailed atomic concentration for each bonding with different UV irradiation times is listed in Table S1.

### Photo oxidative reaction in ceria slurry and its impact on CMP

CeO_2_-based materials have attracted considerable attention for applications in heterogeneous catalysis due to their excellent oxygen exchange capability, related to a facile exchange between Ce^3+^ and Ce^4+^ ions^50–52^. During UV irradiation, ceria provides O' radical with the formation of oxygen vacancy to facilitate the oxidation process of polymers^53^. This mechanism involves two methods for polymer oxidation. The first uses O' obtained from the formation of oxygen vacancy on the surface of a ceria particle and the second is consuming dissolved oxygen in the solution by using ceria particle as a catalyst. These methods are simultaneous, and consumption of oxygen further promotes the formation of oxygen vacancy in ceria, resulting in a virtuous cycle that promotes photo-oxidative degradation of polymers.

**Figure 2:**
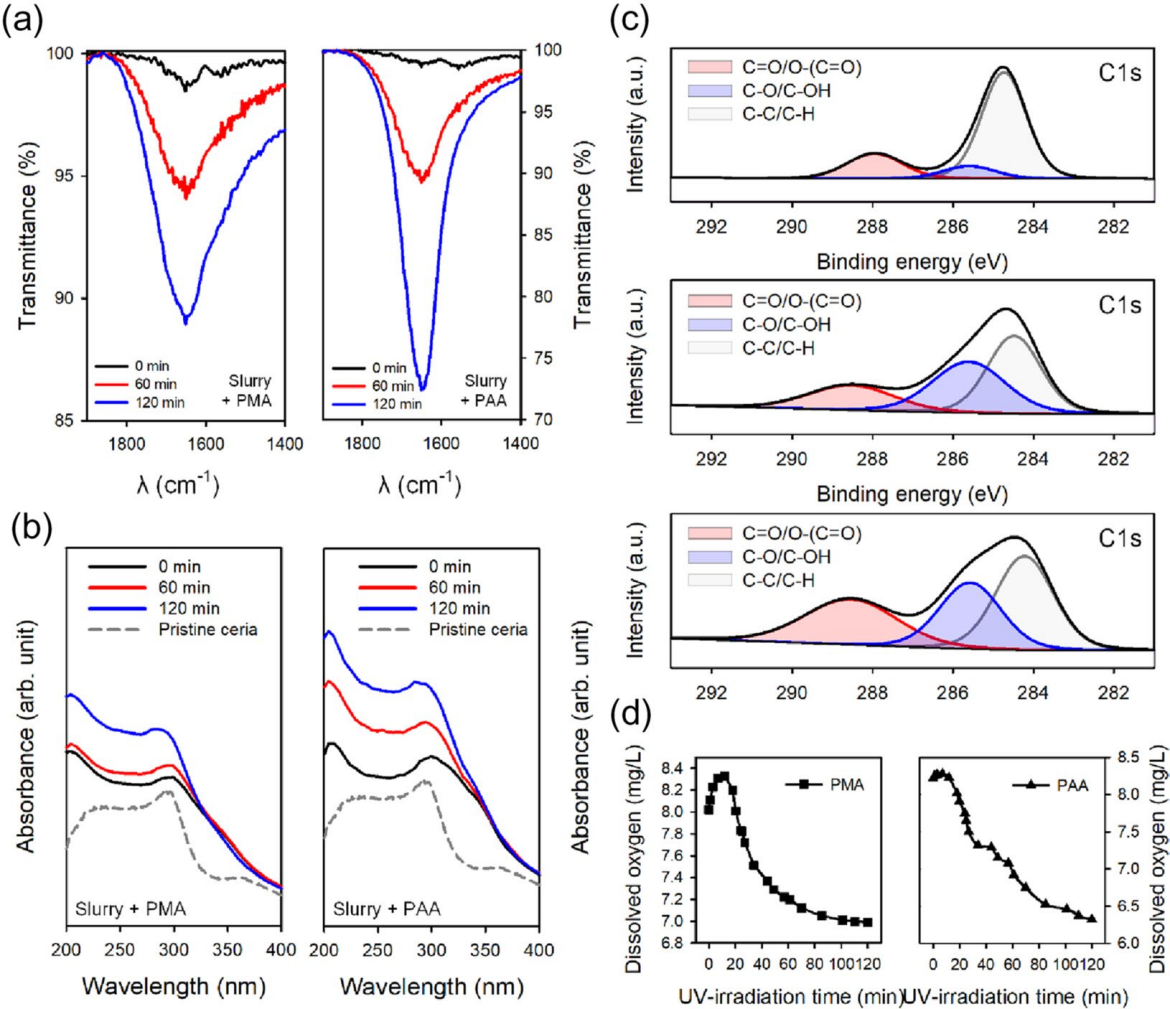
(**a**) Changes in FTIR spectrum of carbonyl band and (**b**) absorbance spectra of ceria slurry with 0.1 wt.% of PMA (left) and PAA (right) during UV-irradiation for 120 min. (**c**) XPS spectra of ceria slurry with 0.1 wt.% of PAA according to UV irradiation time; (top) 0 min, (middle) 60 min, and (bottom) 120 min. (**d**) Changes in dissolved oxygen during UV irradiation on fabricated ceria slurry for 120 min.

**Figure 3:**
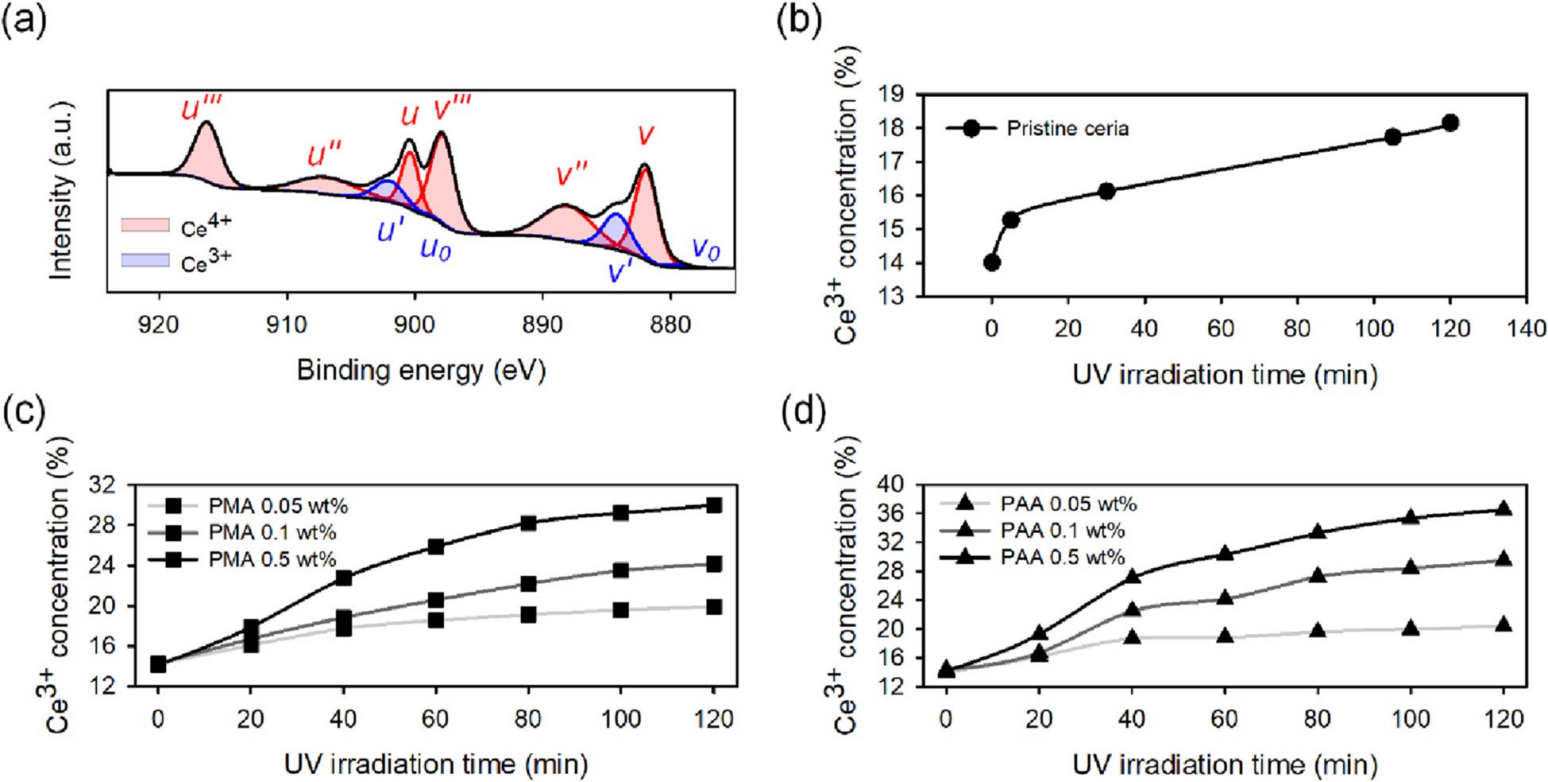
(**a**) XPS spectra of the Ce 3*d* peaks of the CeO_2_ and an increase in the concentration of Ce^3+^ ions according to (**b**) UV irradiation and photo-oxidative degradation of (**c**) PMA and (**d**) PAA at different concentrations.

Figure 2a shows the IR spectra of ceria slurry containing PMA and PAA recorded after different UV irradiation times. The absorbance of the carbonyl band increased significantly because the ceria particles accelerated the photo-oxidation reaction of polyacids. A solution containing only polyacids exhibited the highest absorbance at the beginning of UV irradiation (60 min for PMA and 40 min for PAA). However, a slurry containing ceria nanoparticles showed a further increase in absorbance after UV irradiation for 2 h. This is because the catalytic activity of ceria promoted the photo-oxidation of polyacids, leading to the continuous formation of new types of carbonyl groups in the polymer. UV–vis spectra provide information on the oxidation states of metal ions in a solution. Since the Ce^3+^ and Ce^4+^ ions have absorbance at a different wavelength, the changes in absorbance for each UV region indicate a change in the surface oxidation state of ceria particles^52^. The absorption peaks at 290 and 360 nm are ascribed to Ce^3+^ and Ce^4+^, respectively^51,54^. Figure 2b shows the change in the absorption spectra of ceria slurry with polyacids by UV irradiation. There is no significant change in the absorption peak at 360 nm was observed, however, the peak at 290 nm drastically increased with increasing duration of UV irradiation. In company with each increase in absorbance of polyacids and pristine ceria according to UV irradiation as shown in Fig. 1b and Figure S1, the significant increase in absorbance under 300 nm demonstrates that the formation of the chromophore and Ce^3+^ ions was promoted through UV irradiation. The difference in absorption spectra was not observed only by mixing the polymer in the ceria slurry without UV light, therefore, no chemical reactions or bonds between them occurred in the absence of external energy as shown in Figure S2. Figure 2c shows the C 1*s* peak of PAA contained in the ceria slurry according to each 0, 60, and 120 min of UV irradiation time. When UV light was irradiated on the PAA added in ceria slurry, C=O and C–O were produced at higher rates than that of the only PAA with UV irradiation as listed in Table S2. Comparing to XPS spectra of UV irradiated PAA, the ceria nanoparticles played a significant photocatalytic role to promote the photo-oxidative degradation of polyacids. Therefore, the results of FTIR and UV–vis confirm the increase in Ce^3+^ concentration in ceria slurry via UV irradiation and the photo-oxidative degradation of polyacids. Figure 2d shows that upon addition of PAA and PMA to ceria slurry, the concentration of dissolved oxygen increased slightly up to 10 min of UV irradiation, but decreased gradually with further increase in time. The decrease in the dissolved oxygen is a clear outcome of the photo-oxidation of polyacids. This was the reason for the addition of polyacids to form additional oxygen vacancies on the surface of the ceria particle. In this experiment, PAA consumed more dissolved oxygen for photo-oxidative degradation than that of PMA. From the results of the UV irradiation, the dissolved oxygen in the ceria slurry decreased up to ~23%. The concentration of dissolved oxygen was not lower than 6.3 mg/L because the chamber for UV treatment on slurry was not airtight. The formation of oxygen vacancy via UV irradiation was directly related to the reduction of the surrounding Ce^4+^ ions, and the reduction of CeO_2_ was identified by measuring the Ce^3+^ concentration using XPS. Figure 3a shows the XPS Ce 3*d* spectra of ceria nanoparticles. Ten peaks were observed in the Ce 3*d* valence band, among which the identified peaks were labeled u_0_, u, u′, u′′, and u′′′, corresponding to Ce 3*d*3/2, and v_0_, v, v′, v′′, and v′′′, corresponding to Ce 3*d*5/2. Among these peaks, u′, u_0_, v′, and v_0_ correspond to Ce^3+^ ions, while the others correspond to Ce^4+^ ions. Based on these results, the Ce^3+^ concentration was determined by the ratio of Ce^3+^ to the total as shown by the following Equation (10–12)^55,56^.

10 $$ Area\left( {Ce^{3 + } } \right) = A\left( {u^{\prime}} \right) + A\left( {u0} \right) + A\left( {v^{\prime}} \right) + A\left( {v0} \right) $$

11 $$  Area(Ce^{{4 + }} ) = A\left( u \right) + A\left( {u^{{\prime \prime }} } \right) + A\left( {u^{{\prime \prime \prime }} } \right) + A\left( v \right) + A\left( {v^{{\prime \prime }} } \right) + A\left( {v^{{\prime \prime \prime }} } \right)  $$

12 $$ \% (Ce^{3 + } ) = Area(Ce^{3 + } )/[Area(Ce^{3 + } ) + Area(Ce^{4 + } )] $$

The concentration of Ce^3+^ ions in ceria particles increased from 14% to 18.1% with increasing UV irradiation time, as shown in Fig. 3b. Due to the formation of oxygen vacancies on the surface of the ceria particles by UV light, Ce^4+^ ion was reduced to Ce^3+^. However, the rate of reduction was inhibited by dissolved oxygen. Figure 3c, d shows an increase in Ce^3+^ concentration with increasing concentrations of PMA and PAA additives and increasing UV irradiation time. In this connection, XPS spectra of each prepared slurry before and after UV irradiation for 120 min were shown in Figure S3 and the concentration of Ce^3+^ was listed in Table S3. The addition of polyacids only hardly affects the concentration of Ce^3+^ ions. However, in environments given UV light, the formation of oxygen vacancies on the ceria particles via photo-oxidation of polyacids was promoted, showing a high rate of increase in the concentration of Ce^3+^ ions. The increase in the addition of polyacids allowed the reduction of ceria with higher efficiency, and the PAA had a significant effect on reducing ceria particles than that of PMA. It was observed that photo-degraded PAA consumes more oxygen and has a higher absorbance than that of PMA at the same concentration. This indicates that photo-oxidative degradation might be more susceptible to UV light than that of PMA. Therefore, at the same concentration, PAA always showed a higher reduction efficiency than that of PMA. When 1 wt.% PAA was added and UV irradiation was continued for 120 min, the concentration of Ce^3+^ ions increased from 14.2% to 36.5%.

**Figure 4:**
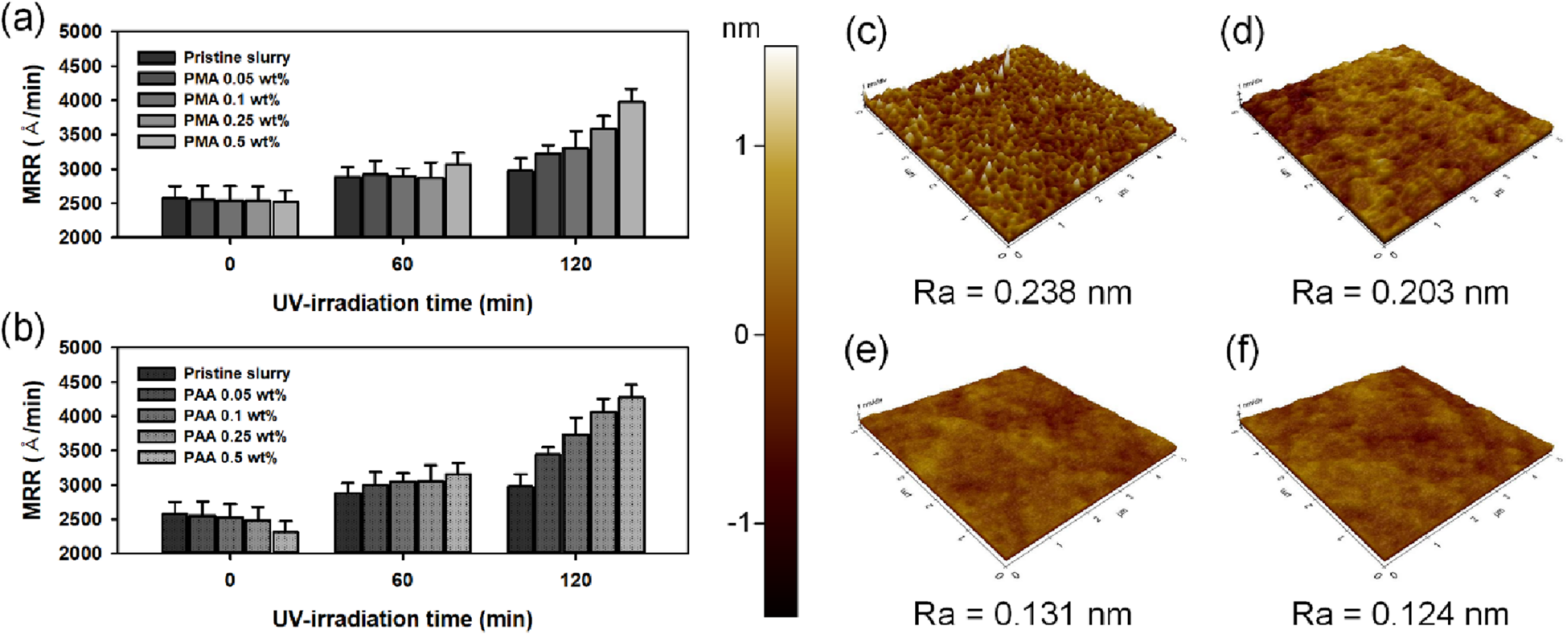
MRR of oxide wafer according to UV irradiation time with varying the concentration of polyacids; (**a**) PMA and (**b**) PAA and following averaged surface roughness (R_a_) of SiO_2_ wafer; (**c**) before polished, (**d**) polished by pristine ceria slurry, (**e**) polished by UV irradiated slurry with 0.5 wt.% of PAA for 1 h and (**f**) for 2 h.

The trivalent state of cerium promotes Ce-O-Si bonding with an enhancement of the interaction between ceria particles and silicon dioxide wafer. Since the formation of Ce-O-Si bonding in an oxide CMP using ceria abrasives is directly related to the material removal rate, high concentrations of Ce^3+^ ions can provide high CMP performance^18,21,57^. Figure 4 shows CMP performance including the removal rate and finished surface roughness of the oxide film using photo-reduced ceria nanoparticles according to UV irradiation time. The CMP results show an increased removal rate upon UV irradiation on pristine ceria slurry. When UV light was irradiated for 120 min, the removal rate was 2973.05 Å/min, which was an increase of 20% compared with that of the removal rate of oxide film using pristine ceria slurry. In general, the addition of a surfactant can lead to a decrease in MRR^58^. Surfactants are adsorbed on particles or wafers to form a passivation layer and reduce MRR by inhibiting mechanical interaction between abrasives and the surface of a wafer. The MRR was hindered by an increase in molecular weight and concentration of surfactants^59–61^. PAA used in this experiment are anionic surfactants and have negative potential in hydrated states. Therefore, it can be easily adsorbed to ceria abrasives exhibiting positive potential, especially in neutral or acidic media, which can interfere with polishing performance^62^. However, in this experiment, the ceria slurry was prepared with a pH of 9 to avoid the problem of adsorption. Since the particles have a negative charge in the basic medium, interference due to an addition of the polyacids during the polishing process was as small as to be insignificant. Therefore, the removal rate was maintained or was slightly decreased when up to 0.5 wt.% of polyacids were added. However, when UV light was irradiated, a higher rate of increase in MRR was obtained than that of pristine ceria slurry. With increasing duration of irradiation, it was observed that a higher removal rate as much as an increase in Ce^3+^ concentration, also a higher MRR was obtained according to the addition of a higher concentration of polyacids. As a result, when UV light was irradiated on ceria slurry containing 0.5 wt.% of polyacids for 120 min, the material removal rate was increased up to 3977.73 Å/min with PMA and up to 4514.05 Å /min with PAA, as shown in Fig. 4a, b. The reactivities of ceria abrasives were enhanced via photo-oxidative degradation of polyacids, resulting in increased CMP performance of ceria slurry by up to 87%. Figure 4c–f shows the AFM images of the surface roughness of the polished wafer. A smooth surface of the polished wafer was obtained with an increase in the time of UV irradiation on the fabricated slurry. The surface roughness of the wafer after CMP is closely related to the dispersibility of the abrasives, which will be described in detail in the following sections. As a result, the performance of high removal rate and smooth surface indicate that the performance of ceria slurry with polyacids was significantly improved through UV irradiation.

### Dispersibility improvement through photo-oxidation

Anionic polymers such as polyacids have been used to disperse positively charged ceria particles^5,8^. When polyacids were added to the slurry, the COOH groups of PAA were deprotonated to negatively charged COO- groups at pH above its pK_a_ of 4.5 and were adsorbed on the positively charged ceria surface via electrostatic attractive interaction and hydrogen bonding, which increased the electrostatic repulsion as well as a steric hindrance between the ceria particles.

However, PAA-coated ceria particles are known to undergo transitions of bridging agglomeration-stable-flocculation depending on their physicochemical conditions such as pH, ionic strength, temperature, and concentration^63^. In this case, the polymer interferes with the interaction among particles, so that they do not come into direct contact with each other, however, it is difficult to confirm that the dispersibility of a solution is good due to the bridging agglomeration of nanoparticles by polymers^35,64^. In this experiment, the ceria particles have negative potential because the pH of the slurry was adjusted to 9^63^. The repulsive force between a polymer and a particle surface becomes strong with increasing pH, which hinders the adsorption of polyacids on the ceria surface. Therefore, in solutions with pH 9, only a small amount of polyacid is adsorbed on the partially positively charged surface of the ceria particle or floating in the solution as a polymer chain state as shown in Figure S4^65–67^. The polymer chain which has the same charged state as colloids could prohibit an agglomeration of particles through electrical repulsion force and steric hindrance^31,62^. Improvements in the dispersibility of a slurry can be defined in terms of three criteria: reductions in the mean diameter of particles, the size distribution width of particles, and the polydispersity index. The polydispersity index is a measure of the heterogeneity of a sample in terms of size^68^.

**Figure 5:**
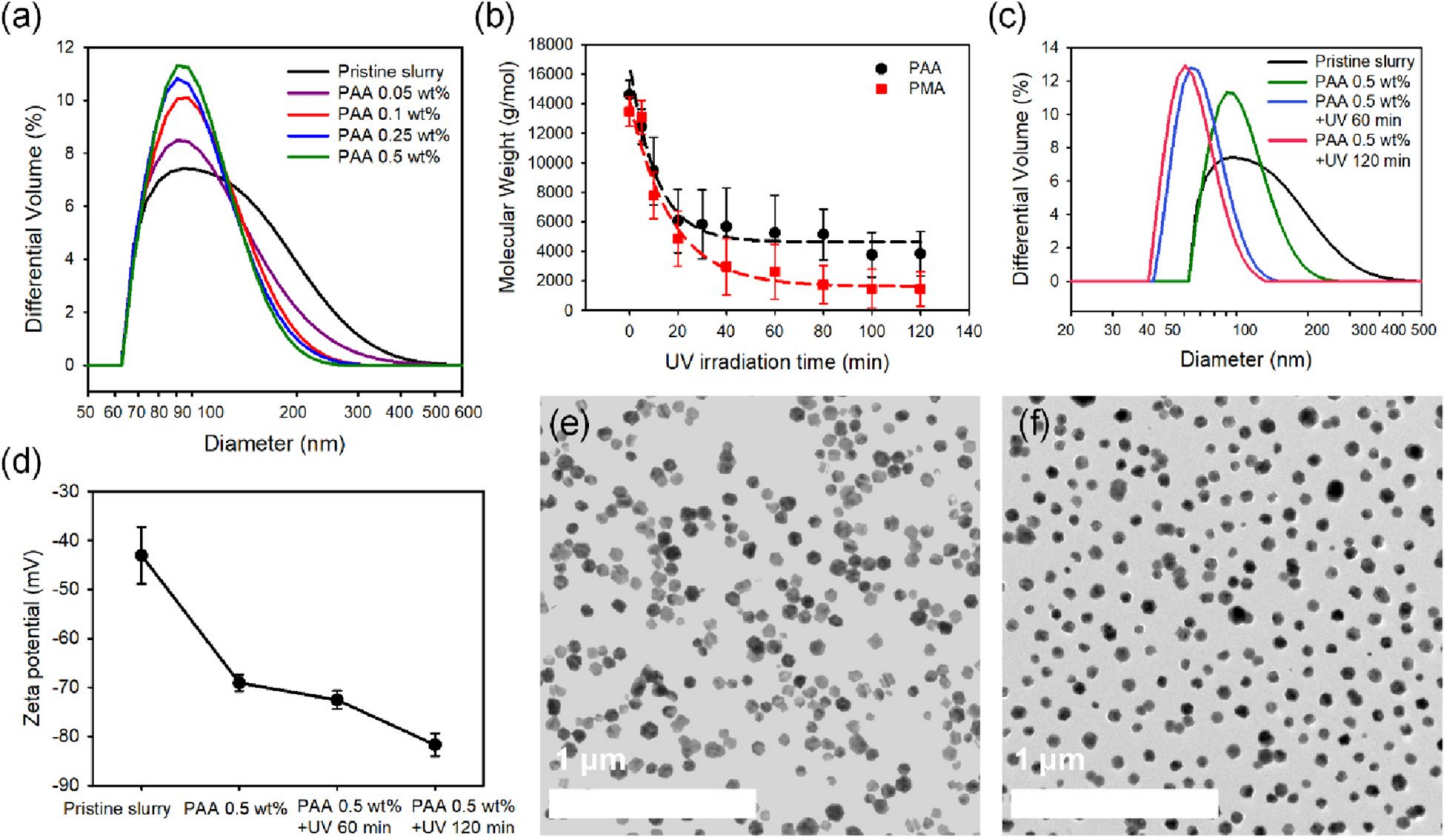
(**a**) Particle size distribution of ceria slurry according to the addition of PAA with various concentrations. The improvement of dispersity through photo-oxidative degradation of polyacids was verified by measuring the changes in (**b**) molecular weight of poly acids due to the photo-oxidative degradation of polyacids, (**c**) particle size distribution, and (**d**) zeta potential of ceria slurry according to UV irradiation time with fixed PAA concentration of 0.5 wt.%. TEM images of (**e**) pristine ceria slurry and (**f**) UV irradiated ceria slurry with polyacids confirms the dispersity of the slurry.

Table 1 lists the mean diameter of ceria particles according to the addition of PAA with various concentrations. There were no significant changes in median diameter (D50) of ceria particles with increasing polymer concentration. However, the decrease in the width of size distribution as shown in Figure 5a and the polydispersity index listed in Table 1 indicate the positive effects of polyacids in improving the dispersity of a slurry. Pristine ceria slurry has low dispersibility, so it has a wide particle size distribution and a large median diameter. The dispersity of pristine ceria slurry was improved with an increase in the concentration of polyacids by enhancing electrostatic repulsion and preventing particle agglomeration^31,32^. In addition, the dispersion effect increases with decreasing length of the polymer chain. The magnitude of the repulsive contribution from depletion effects increases as the concentration increases but decreases as the chain length increases^69–71^. Surfactants with larger molecular weight could not freely move around nanoparticles and effectively adsorb on their surface so that the dispersibility of the slurry couldn’t be improved efficiently. As UV irradiation time increases, polymers consume oxygen and undergo photo-oxidative degradation, resulting in a decrease in molecular weight as shown in Fig. 5b. Since polymers with shorter chain length tend to reside in solutions compared with polymers with longer chains, bridging agglomeration was inhibited and the free volume between particles and polymers was reduced to improve the stability of nanoparticles^72^. Furthermore, the presence of polymer chains of multiple lengths can improve dispersibility. Photo-oxidized polymers have a wider distribution of molecular weight, although the average molecular weight distribution is lower than that of the conventional refined molecular weight distribution. Therefore, the adsorbed polymer forms a rough surfactant layer on the surface of the particle. Consequently, the dispersity of ceria slurry was improved with increasing UV irradiation time due to photo-oxidative degradation of polyacids as shown in Fig. 5c. The rough surfactant layer is responsible for the improved dispersion stability through the enhanced solvent–surfactant interaction and decreased interactions between the interparticle surfactants^73,74^. Changes in zeta potential over chain length also played a significant role in the dispersibility of particles. Figure 5d shows the variation in the zeta potential of ceria slurry containing 0.5 wt.% of PAA with UV irradiation time. The zeta potential of pristine ceria was −43.07 mV, and the electrokinetic potential was enhanced to −82.73 mV with increasing UV irradiation time. The chain expansion occurs due to the reactive electronic interaction caused by negative potential between particles and adsorbed polymers in a solution with a pH of 9. This enhances the shear plane potential, thus achieving a high absolute value of zeta potential. Therefore, the electrical repulsion force between particles and polymers increases, improving dispersibility, as UV irradiation time increases. TEM images shown in Fig. 5e, f confirmed that the dispersibility of ceria nanoparticles was enhanced upon the addition of PAA with UV irradiation for 2 h.

**Table 1:** The particle size distribution and polydispersity index of ceria slurry according to the concentration of PAA and the duration of UV irradiation.

Cumulant size	Pristine ceria	PAA concentration (wt.%)				PAA 0.1 wt.% + UV irradiation time (min)	
		0.05	0.1	0.25	0.5	60	120
D10 (nm)	72.7	75.8	71.9	72.5	58.8	51.9	48.4
D50 (nm)	115	102	96.5	96.3	83.6	66.4	61.7
D90 (nm)	213.2	151.3	146.1	140.1	137.3	90.8	84.3
PI	0.206	0.077	0.098	0.097	0.086	0.085	0.060

The dispersity of abrasive is closely related to the surface roughness of polished wafers^75–77^. Since a slurry with low dispersity forms a significant amount of agglomerated large particles, it is considerably affected by mechanical abrasion and the surface of the polished wafer becomes rough. Based on the fully plastic deformation and the abrasive indentation assumption, the indentation depth (*∆*) of the spherical abrasives into the wafer can be derived as follows^78^

13 $$ \Delta = \frac{2F}{{\pi xH_{w} }} $$

where *x* is the diameter of the abrasives and *H*_*w*_ is the hardness of the wafer. *F* is the force applied on a single abrasive, which can be calculated as follows.

14 $$ F = 0.25\pi P_{c} x^{2} $$

where *P*_*c*_ is the contact pressure from a specified contact area of the pad asperity. According to Eqs. (13, 14), the indentation depth is proportional to the diameter of abrasives. Because the indented mark is the result of abrasion on the surface of the wafer by the particles, a decrease in the particle size indicates a decrease in the indentation depth, which in turn leads to reduced surface roughness. Yang et al. also verified an increase in the surface roughness as a function of an increase in the size of the ceria nanoparticles^75^. In this experiment, the dispersity of ceria slurry was improved with the addition of polyacids and their photo-oxidative degradation by irradiating UV light. After UV treatment with PAA, the D50 of the particle size distribution decreased from 115 nm to 61.7 nm. As a result, a smooth surface of polished wafer could be obtained with an increase in the UV irradiation time as shown in Fig. 4c–f. The average roughness decreased from 0.238 nm to 0.124 nm when the UV light was irradiated on the ceria slurry for 2 h. A smooth surface was obtained due to the photo-oxidative degradation of polyacids, although the MRR drastically increased.

## Conclusion

In this study, photoreactions of ceria and polyacids were performed in a basic solution to improve the CMP performance of ceria slurry. UV irradiation on ceria lowers the oxygen vacancy formation energy, increasing the concentration of Ce^3+^ ions on the surface of the ceria nanoparticles. Since Ce^3+^ ion is an active site involved in material removal by forming a Ce-O-Si bond with an oxide wafer, an increase in Ce^3+^ concentration leads to an increase in MRR. Simultaneously, the photo-oxidative degradation reaction of polyacids contributed to the improvement of the polishing performance of the ceria abrasives in two ways. First, the degradation of polymers by UV light significantly affected the formation of Ce^3+^ ions on the surface of the ceria particles by reducing the contents of dissolved oxygen, which inhibited the reoxidation of ceria particles and facilitated the formation of oxygen vacancies, resulting in a significant improvement in MRR. The MRR increased with increasing polyacid concentration to 0.5 wt.% and increasing duration of UV irradiation time to 2 h. Second, during the photo-degradation process, the molecular weight of polyacids was reduced and shortened polymer chains reside in the solution to enhance dispersity of slurry by strengthening the electrostatic repulsive force between particles. The ceria slurry, which has improved dispersibility due to the degradation of polyacids, was used to polish the wafer surface comparatively smoothly, resulting in low average roughness. Therefore, the CMP performance, in terms of removal rate and surface roughness, was significantly improved by photo-reduction of ceria and photo-oxidative degradation of polyacids induced by UV light irradiation.

## Methods

### UV irradiation on ceria slurries

In this experiment, 60-nm commercial ceria abrasive was used to polish the oxide film. The nanoparticles were diluted in deionized (DI) water to obtain a concentration of 0.5 wt.% of solid contents. Two types of polyacid solutions, containing either PAA (Wako, Japan) or PMA (Sigma Aldrich, USA) (average molecular weight 10,000 g/mol), were used to achieve particle dispersion and photo-oxidation effects. Both polymers have a similar structure and are anionic surfactants that exhibit a negative potential when dissolved in water. In addition, since the ceria particles have a negative potential in a basic solution, KOH was added to the ceria slurry to obtain a pH of 9, thus preventing the adsorption of polyacids on particles and inhibiting interactions between particles and the wafer surface. Moreover, the polymer concentration was maintained at a level lower than that of the solid contents to prevent the polymer from impeding the mechanical abrasion between the abrasives and the wafer.

A closed chamber was fabricated to ensure effective irradiation of UV light on the ceria slurry. An Al foil was used to cover the inner wall of the chamber to prevent leakage of light and increase the efficiency of irradiation. A magnetic stirrer was installed at the bottom of the chamber, which stirred the ceria slurry at 400 rpm to ensure uniform UV light exposure. A UV light source was installed on the wall above the chamber. It was fixed at a distance of 3.5 cm apart from the surface when 500 mL of the slurry was placed in the beaker. The wavelength of the light generated by the UV-light source was 254 nm at 6 W, and the intensity of UV light on the surface of the solution was 489 mW/cm^2^^25^. Since the light of wavelength lower than 254 nm can provide photon energy greater than that of the bandgap of the ceria particles (3–3.6 eV^79^), the separation of the electron-hole pair from the valance band of ceria decreases the oxygen vacancy formation energy from 1.16 eV to -0.64 eV in the presence of UV light^25^. With the lowered oxygen vacancy formation energy, the electrons and holes generated from the UV irradiation reduce Ce^4+^ to Ce^3+^ and the holes oxidize O^2−^ anions^24,29^. Additionally, it can provide sufficient energy to generate chain scission of polymers.

### Confirmation of photo-oxidative degradation of polyacids

Polyacids under the exposure of UV light in the chamber form chromophores such as ethylene bonds and carbonyl groups, during photo-degradation. The change in the intensity of absorbance depends upon the concentration of chromophores. The change in the absorption spectra was measured by UV–visible spectroscopy (UV-3600, SHIMADZU, Japan). The photo-oxidation processes were confirmed by Fourier-transform infrared (FTIR) spectroscopy (IFS-66/2, Bruker, USA). The total carbonyl group contents were calculated from the area of the peaks in the range of 1600–1800 cm^−1^. The photo-oxidative degradation process of polyacids in ceria slurry was also confirmed by UV–visible spectroscopy and FTIR spectroscopy. Moreover, the decrease in the concentration of dissolved oxygen due to photo-oxidation of polyacids was measured in real-time using a dissolved oxygen meter with a microsensor (Oxyscan 300, UMS GmbH & CO. KG, Germany). It was also confirmed that the photo-oxidative degradation caused chain scission in polyacids by measuring the changes in the molecular weight of polyacids through a molecular weight analyzer with static light scattering (SLS) method (ELSZ-2000, Otsuka Electronics Co.). The photo-reduction of ceria particles enhanced by photo-oxidation of polymer can be verified as a change in the concentration of Ce^3+^ ions in the ceria particles. The concentration of Ce^3+^ ions, which is the final product affecting CMP performance, was determined by X-ray photoelectron spectroscopy (XPS; ESCALAB 250, Thermo Fisher, USA).

### Chemical mechanical polishing (CMP)

For the CMP process, the oxide wafers were polished on a rotary-type CMP machine (Poly-400, G&P Technology, Korea) with a polyurethane polishing pad (IC1010, Dupont, USA). The polishing pad was conditioned before polishing for 30 min to obtain a uniform surface. The mechanical conditioning process was performed simultaneously while polishing the wafer with a load force of 3 kgf to ensure that the pores are not clogged and to prevent changes in CMP performance. CMP slurry was prepared by mixing ceria nanoparticles, polyacids, and pH adjuster with DI water. The fabricated slurry was fixed at 0.5 wt.% of solid contents and pH 9, and the initial dissolved oxygen is 8.1 mg/L on average. The prepared CMP slurry is irradiated to UV light for a specific time in the chamber with continuous stirring and then directly supplied to the CMP process. The slurry is supplied directly from the chamber to the CMP equipment still under UV irradiation. The load pressure applied on the wafer was 3 psi. The rotation speed of the platen was 93 rpm and the rotation speed of the wafer head was 87 rpm. Table 2 provides additional information about the experimental conditions for polishing. The material removal rate (MRR) of the polished oxide film was obtained by measuring the difference between the thickness of the oxide film before and after CMP using a reflectometer (ST5030-SL, K-MAC Co., Korea). Finally, the final surface of the polished wafer was observed using atomic force spectroscopy (AFM; NX10, Park systems, Korea).

**Table 2:** CMP condition.

Parameter	Condition
Material	1-μm-thick SiO_2_ film on four-inch wafer
Pad	IC1010/Suba IV
Down pressure on the wafer	4 psi
Down force on conditioner	3 kgf
Pad rotation speed	93 rpm
Wafer rotation speed	87 rpm
Slurry flow rate	120 mL/min
Process time	1 min

### Confirmation of dispersity in ceria slurry

The dispersion of nano-abrasives in the slurry is an important factor that affects the CMP performance, in terms of surface defects including surface roughness or scratches^80^. The dispersity of particles can be determined from changes in the average particle size and the width of particle size distribution^65,81^. A particle sizer was used to measure the mean particle size and size distribution by the dynamic light scattering (DLS) method. The addition of polyacids and the effect of photo-degraded polyacids on the dispersion of the ceria particles were confirmed using the D10, D50, and D90 values of the cumulative particle size to quantify both particle size and size distribution. Furthermore, the changes in the zeta potential of the ceria slurry due to the decomposition and ionization of polymers were measured. Finally, the agglomeration of particles according to dispersibility was visually identified using transmission electron microscopy (TEM; JEM-2100F, JEOL, Japan).

## Supplementary Information

Supplementary Information.

## Data Availability

All relevant data are within the paper.
