# Supplementary material for: Photo-oxidative degradation of polyacids derived ceria nanoparticle modulation for chemical mechanical polishing
Source: Sci Rep. 2022 Jan 31;12:1613. doi: 10.1038/s41598-021-03866-9 (PMC8803865; doi:10.1038/s41598-021-03866-9)
Supplement: Supplementary file 1 — Supplementary Information. [file 41598_2021_3866_MOESM1_ESM.docx]

Photo-oxidative Degradation of Polyacids derived Ceria Nanoparticle Modulation for Chemical Mechanical

# Eungchul Kim1, Jiah Hong1, Hyunho Seok2, and Taesung Kim1,2,*

1 Sungkyunkwan University, School of Mechanical Engineering, Suwon, 16419, South Korea.

2 Sungkyunkwan University, SKKU Advanced Institute of Nanotechnology (SAINT), Suwon, 16419, South Korea.

* [tkim@skku.edu](mailto:tkim@skku.edu)

# Supplementary

**Figure S1.** Absorbance of pristine ceria before/after UV irradiation.


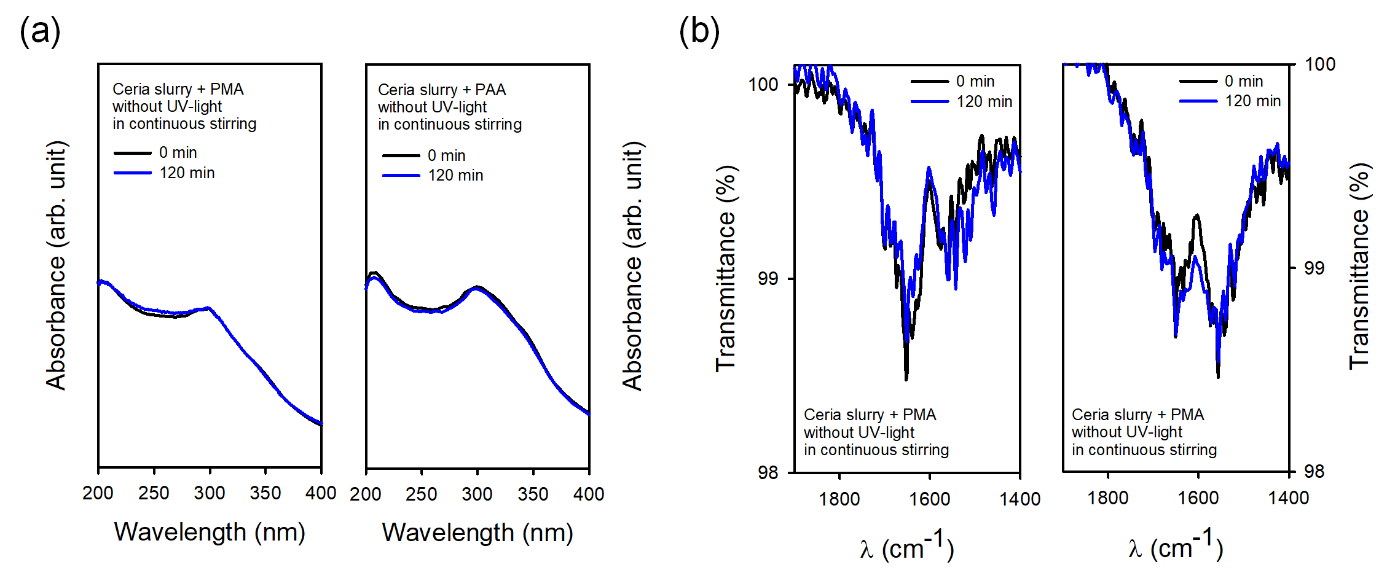


**Figure S2.** (a) Absorbance spectra and (b) FTIR spectrum of ceria slurry containing PMA (left) and PAA (right) without UV-light at continuous stirring in dark for 120 min.


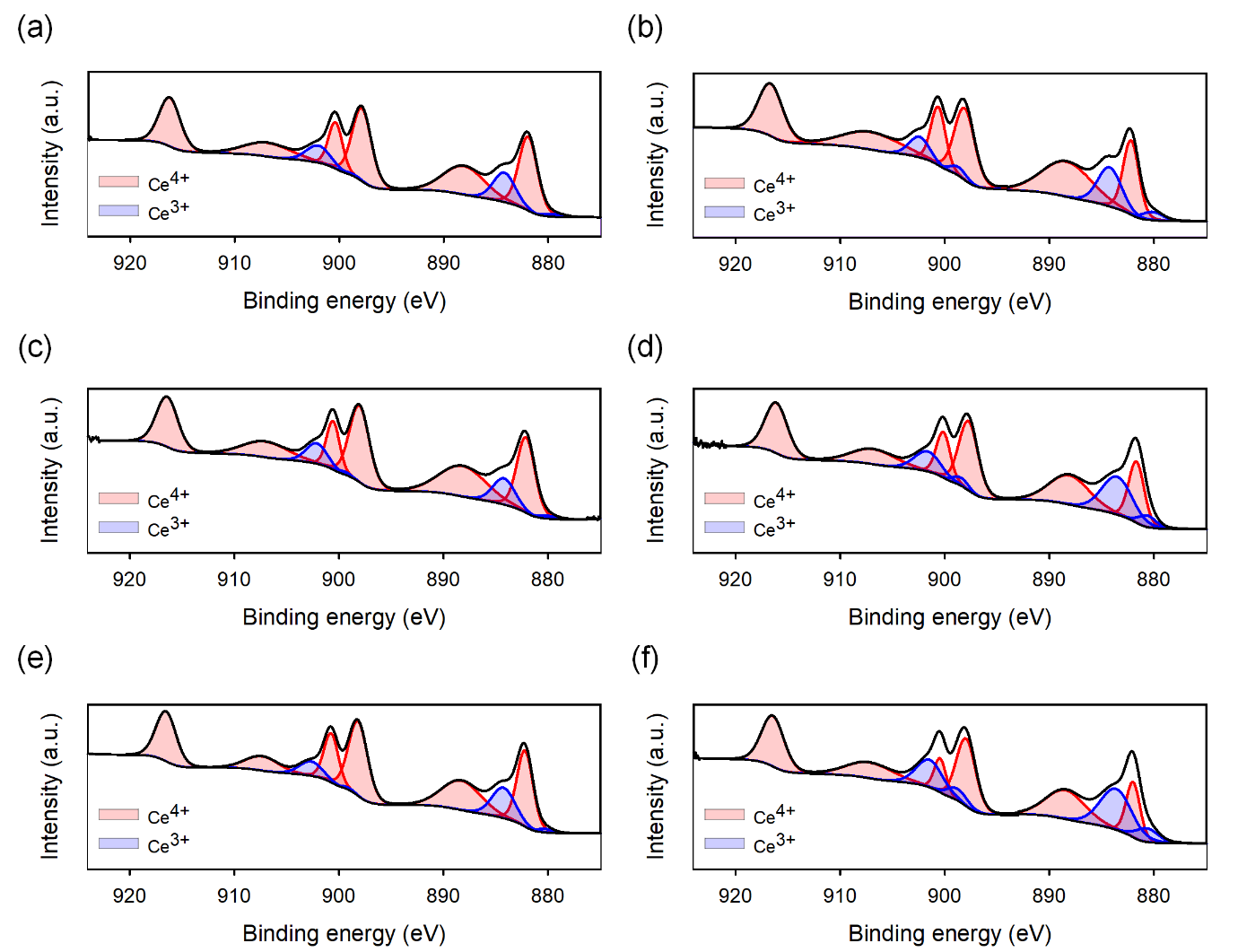


**Figure S3.** XPS Ce 3*d* spectra of pristine ceria slurry with (a) before and (b) after, of ceria+PMA slurry (c) before and (d) after, and of ceria+PAA slurry (e) before and (f) after UV irradiation for 120 min.


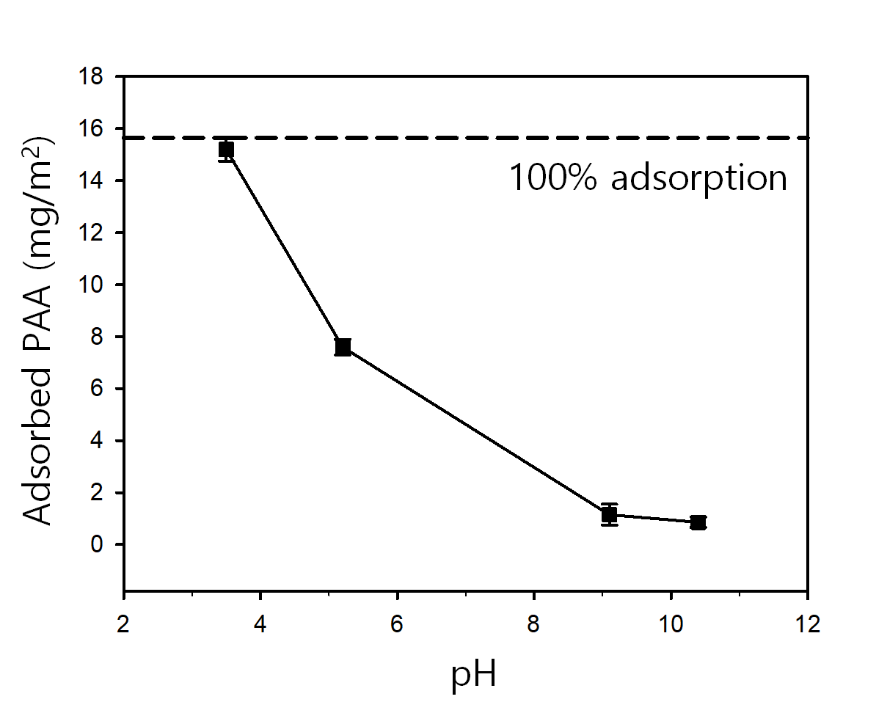


**Figure S4.** Adsorption isotherms for PAA on ceria particles according to slurry pH. The adsorption of polyacids on ceria nanoparticles was determined by the solution-depletion method. The 0.5 wt% of ceria nanoparticles and 0.1 wt% of PAA were diluted in deionized (DI) water at a pH range of 3 to 11. The slurry then stabilized by a ball mill equipment at 200 rpm for 4 hours to equilibrate adsorption of PAA on ceria nanoparticles. After equilibration, the suspensions were centrifuged at 4000 rpm for 30 min to separate the ceria particle, and the supernatant was filtered using a 0.1 mm syringe filter (PVDF, Whatman, England).The total amount of organic carbon in the supernatant was measured by the TOC analyzer (M9, SUEZ, France). The TOC value for the powder blank was first subtracted from the suspension supernatant value to obtain residual concentration. The initial polymer concentration was derived from the control sample that contained dried ceria powder without PAA, and the amount of adsorbed polymer was taken as the difference between the initial and residual concentrations.

| Bonding | Binding  energy (eV) | Atomic concentration with different UV irradiation time (%) | | |
| --- | --- | --- | --- | --- |
|  |  | 0 min | 60 min | 120 min |
| C=O | 288.2 | 19.97 | 32.8 | 29.25 |
| C-O | 285.8 | 4.35 | 7.15 | 22.03 |
| C-C | 284.4 | 75.68 | 60.05 | 48.72 |

**Table S1.** The atomic concentration of C 1*s* spectra of PAA according to UV irradiation time.

| Bonding | Binding  energy (eV) | Atomic concentration with different UV irradiation time (%) | | |
| --- | --- | --- | --- | --- |
|  |  | 0 min | 60 min | 120 min |
| C=O | 288.3 | 20.48 | 21.27 | 32.59 |
| C-O | 285.6 | 9.71 | 37.25 | 32.43 |
| C-C | 284.4 | 69.8 | 41.48 | 34.97 |

**Table S2.** The atomic concentration of C 1*s* spectra of PAA contained in ceria slurry according to UV irradiation time.

| Material | Ce^3+^ concentration (%) | Material | Ce^3+^ concentration (%) |
| --- | --- | --- | --- |
| Pristine ceria | 14.21 | Pristine ceria + UV | 18.15 |
| Ceria + PMA | 14.27 | Ceria + PMA + UV | 24.17 |
| Ceria + PAA | 14.38 | Ceria + PAA + UV | 29.56 |

**Table S3.** Ce^3+^ concentration of fabricated slurry according to UV irradiation for 120 min.
